# Can Land Policy Promote Farmers’ Subjective Well-Being? A Study on Withdrawal from Rural Homesteads in Jinjiang, China

**DOI:** 10.3390/ijerph19127414

**Published:** 2022-06-16

**Authors:** Fachao Liang, Zehua Wang, Sheng-Hau Lin

**Affiliations:** 1 School of Political and Public Administration, Huaqiao University, Quanzhou 362021, China; fcliang@hqu.edu.cn (F.L.); 20013031009@stu.hqu.edu.cn (Z.W.); 2 Department of Public Administration, Law School, Ningbo University, Ningbo 315211, China

**Keywords:** withdrawal from rural homestead (WRH), happiness, subjective well-being (SWB), rural revitalization, sustainable rural development

## Abstract

Urbanization and aging populations are threatening the sustainability of rural development around the world. Improving the happiness of rural residents is closely related not only to rural development but also to the harmony and stability of a country. Sustainable development has become an important strategy for China’s rural areas. Although withdrawal from rural homesteads is an important issue in rural land policy, few researchers have examined the determinants of the subjective well-being of farmers following withdrawal. The current paper investigated 315 rural residents under three models of the “withdrawal from homestead” policy in Jinjiang City, Fujian Province, China. The application of the orderly probit model revealed how satisfaction with economic, social, environment, cultural, and policy factors impacted their subjective well-being. The pooled results showed that satisfaction with cultural and policy factors had no significant impact; however, the other aspects significantly promoted their subjective well-being. The empirical model with interaction terms indicated the significant positive impact of economic, environmental, and social factors on subjective well-being under the index replacement model, while only environment and social factors exerted a significant positive impact under the asset replacement and monetary compensation models. Corresponding policy implications are discussed.

## 1. Introduction

Urbanization and aging populations worldwide have increased the occurrence of rural hollowing, which refers to the neglect and vacancy of rural dwellings [1]. Withdrawal from rural homesteads (WRH) is a land policy which attempts to organize idle and abandoned homesteads to optimize the use of rural areas to provide residents with living and production spaces to meet basic needs [2], including the happiness of local residents. Algan [3] pointed out that happiness reflects an individual’s satisfaction with life as well as the quality of the social system in which they live. Research shows that rural residents are happier than urban residents [4], likely due to high levels of urbanization in urban areas and contemporary social networking in rural areas [5]. Improving the happiness of rural residents is closely related to rural development; however, it is also related to the harmony, stability, and sustainable development of a country [6]. Exploring this issue is crucial to achieving the third goal of the 17 Sustainable Development Goals (SDGs)—ensuring healthy living and promoting life well-being [7].

In the context of rapid urbanization in developing countries such as China or India, the livelihood of rural residents, which depends on access to productive land, is under threat. The impact of the transfer of agricultural land is an important index measuring whether transfer behavior brings positive benefits. Land-lost rural residents have become a significant focus of studies on happiness [8]. More and more studies in developing countries have been focusing on the happiness of rural residents and its determinants after farmland expropriation or lease [9,10,11,12]. However, rarely has research focused on the relationship between WRH and the happiness of farmers in rural China. The term ‘homestead’ refers to land allocated by Chinese townships or village collectives to residents for the construction of housing. WRH offers money or new houses as compensation to residents for giving up their right to rural homesteads [13]. Homesteads play a decisive role in residents’ livelihoods and rural society. The WRH policy is a key strategy to achieve “rural revitalization” based on the premise of perfecting integrated rural development and enhancing rural sustainability [14].

The close connection between WRH policy and sustainable rural development lies in activating existing land resources of farmers who have withdrawn and enhancing the life cycle of farmers’ houses by redeveloping or renovating existing houses which are not being used efficiently. Systematic re-planning of the countryside has been adopted as the model that will ensure the sustainable development of China through improving living environments and maximizing idle land resources. Rural residents face both favorable and unfavorable impacts after WRH. These impacts involve land resources, human resources, and personal wealth [15]. Farmers benefit from WRH policy through the improvement of idle housing and rural living environments. This results in higher satisfaction with the policy, which is conducive to the expansion and implementation of the policy. However, the implementation of WRH policy may encourage rural residents to relocate to more urbanized areas to experience more convenient living conditions and better access to facilities that promote their health and well-being. Local governments can re-arrange rural land after WRH to provide public services needed to optimize land use [16]. Investigating farmers’ perceptions following the implementation of WRH enables us to examine the policy effects of WRH. Residents may suffer from psychological, cultural, and social exclusion due to a lack of social and economic security, conflicts with surrounding communities related to employment opportunities, and an inability to integrate into urban life. While many studies have examined the determinants of WRH from a decision-making perspective [17,18,19,20], the subjective well-being (SWB) of rural residents following WRH has rarely been investigated.

Are rural residents happy after WRH? What aspects of SWB do they experience? As different WRH pilots are implemented in various parts of rural China, different WRH models have different practical effects, such as monetary compensation or asset allocation. Are there differences in SWB under different WRH models? These are the questions asked in this paper. Our results contribute to the literature on WRH and the life quality of rural residents, specifically in helping to understand the perception of residents of WRH policy. Rural residents represent a large group in China, and with current trends of urbanization, this group has an important role to play in realizing the integrated development of urban and rural areas [21]. The Chinese experience in WRH policy could serve to help other developing countries in the design of rural land policies.

The purpose of this study was to determine the perspective of rural residents in Jinjiang City, where China’s first WRH pilot was conducted. We aimed to explore the satisfaction of rural residents in different dimensions and how to increase SWB after WRH as well as further explore the impacts of different WRH modes. The remainder of this paper is arranged as follows: Section 2 presents our review of relevant literature and hypothesis development. Section 3 introduces the research method in detail. Results are discussed in Section 4. Finally, Section 5 provides a brief conclusion and policy recommendations.

## 2. Literature Review and Hypothesis Development

The pursuit of happiness is undertaken by individuals in every culture. Happiness has become an integral part of discussions in sociology, psychology, and economics [22]. The measure of happiness can be divided into objective well-being (OWB) and subjective well-being (SWB). OWB comprises quantifiable living standards. SWB is individuals’ self-assessment of their own living conditions [23,24]. SWB is one of the most important measures of quality of life, and “happiness” and “life satisfaction” are often used interchangeably within the concept of SWB [25]. SWB is measured by collecting personal perceptions and emotional states as well as data on external environmental elements [26].

In recent decades, SWB has received increasing attention from the academic community. The representative “happiness paradox” [27] and the recent “rural happiness paradox” [28,29,30] have promoted this interest. Income [30,31], health [32,33], education level [34], and social capital [35] have all been proven as determinants of rural residents’ SWB.

The main livelihood capital of farmers is land, and empirical studies have found that land acquisition prompts declining health and SWB [36]. Various factors such as unfair or unreasonable land compensation or resettlement policies, social security systems, and salaried employment can also reduce the SWB of rural residents. However, improving community accessibility as well as public facilities and services can have a positive effect on SWB after land acquisition. Qiu [37] found that social capital factors such as trust and kinship increase SWB after farmland is rented out in India. In that study, diversified income sources and consumption patterns, urbanized infrastructure, and land acquisition compensation significantly increased the satisfaction of land-lost residents, thereby promoting their happiness. 

**Figure 1:**
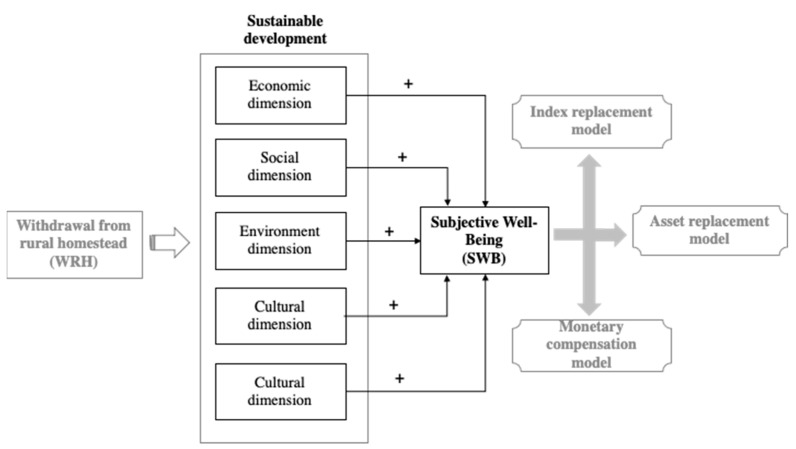
Research framework. **Notes:** Drawn by the authors.

Since the implementation of reform policy in 1978, the Chinese government has achieved considerable economic development but faces serious threats to sustainable rural development, including a shortage of infrastructure investment, poor industrial development, a weak economic foundation, a widening gap between the rich and the poor, and aging residents [38]. Attempts to address these issues resulted in the “Rural Revitalization Strategy”, which was initiated in 2017 and subsequently became the “Rural Revitalization Promotion Law” in 2021. Within this context, WRH is regarded as a key policy to achieve sustainable rural development [39]. In contrast to traditional farmland, homesteads fulfill more diversified functions. They are used not only for farming and production but also for residential and social purposes. This research explores two hypotheses regarding the mechanisms underlying the happiness of farmers after WRH. A research framework is presented in Figure 1.

Rural communities must provide various services for rural people, such as hospitals, schools, and shops [40]. Once abandoned homesteads are withdrawn, the local government can reorganize the land and initiate internal transformation. Some land is cultivated, thereby increasing food production. Local governments can also optimize the land-use structure by rearranging public service facilities and increasing the area dedicated to ecological land and green spaces [16]. Accessibility to rural facilities and services has been shown to affect the SWB of rural residents [5,41]. However, unfair or unreasonable land compensation and resettlement policies regarding WRH may cause dissatisfaction among land-lost residents [8,42]. Moreover, problems with the social security system can cause a decrease in life satisfaction. Culture should also be regarded as an important determinant of SWB. In response to rapid development and the gradual decline in traditional cultural awareness, rural areas are facing difficulties regarding cultural preservation. For example, the SWB of rural residents affected by culture is lower than that of urban residents in Italy from Viganó’s research [27]. We therefore formulated our first hypothesis:

**Hypothesis** **1** **(H1).** *After WRH, the satisfaction of rural residents with economic, social, environmental, and cultural aspects as well as with policy (particularly regarding compensation) positively affects their SWB*.

Different WRH models are carried out in various parts of China, resulting in different impacts on the rural community. Jinjiang City, one of the earliest WRH pilots in China, has implemented three models: asset replacement, index replacement, and monetary compensation [43]. In the asset replacement model, rural identities change from rural household registration to urban household registration, with newly planned urban residences. Centralized, resettled residents will belong to the same social security system as local citizens and gain access to better public facilities [44]; however, they may face economic risks under this model. In the index replacement model, residents retain their household registration and land. Residents therefore retain livelihood capital but are concentrated in collectively built townhouses or high-rise resettlement houses. The rural government is able to re-plan the use of rural land to improve public facilities while preserving the local ethnic culture, clan concepts, and historical heritage. In the monetary compensation model, most residents receive a one-time monetary compensation or urban housing but lose their rural household registration. Local governments then perform land consolidation [45]. The literature suggests that rural residents under the index replacement model are likely to experience higher SWB. This brings us to our second hypothesis:

**Hypothesis** **2** **(H2).** *The satisfaction of residents with economic, society, environment, and cultural factors as well as with policy has differing impacts on their SWB under different WRH models. In particular, the impact of these factors under the index replacement model is more significant*.

## 3. Methods and Data

### 3.1. Study Areas and Sampling Criteria

**Figure 2:**
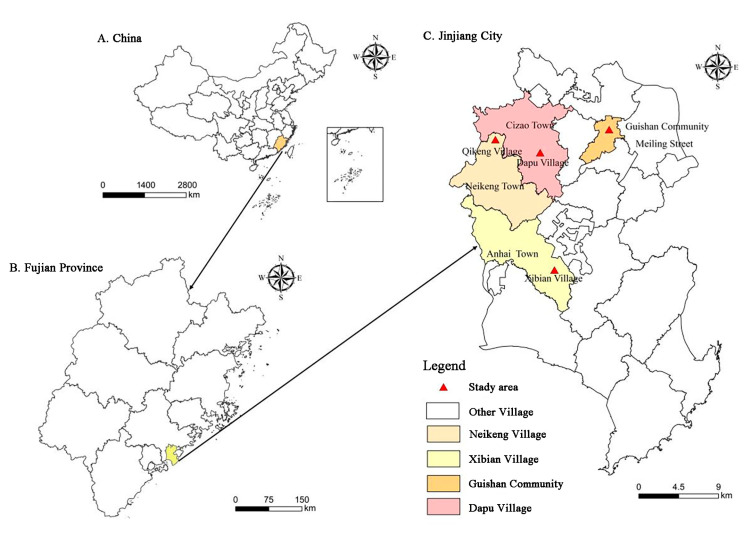
Map of study areas. **Notes:** Maps were collected from Easyearth (http://www.easyearth.com.cn/#/EasyEarthIndex, accessed on 12 June 2022) and the figure was drawn by the authors.

Jinjiang City in Quanzhou, China, is one of 15 pilot cities for WRH policy. It is also the only selected pilot area in Fujian Province. Reform aimed at realizing the intensive use of rural-land resources was launched in 2015. This paper selected four villages representing different WRH policy models in Jinjiang: Qiekeng Village (under index replacement), Dapu Village (under index replacement), Guishan Village (under asset replacement), and Xibian Village (under monetary compensation). As an important representative of the index replacement model, Qiekeng Village was in the first batch of 8 pilot villages for WRH policy in 2015, and Dapu Village was selected for the second batch of policy demonstrations in 2016 based on the base of Qiekeng Village model. Guishan Village is also representative of the asset replacement model of the first batch of pilot villages. Xibian Village is a special case of WRH policy. This area is representative of monetized WRH because it received a large amount of funds donated by local overseas Chinese, so it is also included in the study area. A map of these study areas is presented in Figure 2.

Table 1 summarizes the context and content of WRH policy for the four villages. The WRH policy of Qiekeng Village includes the establishment of village councils, the repair of old buildings, and paid withdrawal from homesteads. It achieved the expected effect of guaranteeing farmers’ housing rights and revitalizing the stock of homestead resources. In addition to unified planning under the leadership of the village committee, the WRH policy of Dapu Village emphasized full communication with the villagers during implementation. Guishan community implemented the asset replacement model, providing villagers with different options after WRH, such as resettlement housing, commercial office buildings, or storefronts, to achieve diversified asset replacement. Overseas Chinese investment and village collective leadership were key factors for Xibian Village in the promotion of WRH policy. Farmers obtained compensation for their land and houses by referring to market prices.

The farmers in these villages were the subjects of this study. WRH policy affects not only individual farmers, but also other co-residents; however, considering the operability of the survey, the main owner of the homestead was the object of the survey. Sampling criteria included the following two conditions: participation in the relevant activities of WRH policy and ownership of the property rights of the existing homestead. Considering the existing population size and limited research time, we applied stratified sampling to distribute 359 questionnaires, of which 100 questionnaires were distributed in Qiekeng Village and Dapu Village, 80 questionnaires were distributed in Guishan Village, and 79 questionnaires were distributed in Xibian Village. The survey was conducted from September 2020 to April 2021. A total of 315 valid questionnaires were recovered, representing a recovery rate of 89.75%, with a validity rate of 87.74%. We recovered 81 samples from Qiekeng Village and 80 samples from Dapu Village (representing the income index replacement model), 62 shares from Guishan Village (representing the asset replacement model), and 62 shares from Xibian Village (representing the monetary compensation model).

### 3.2. Measurement

This study designed a questionnaire in Chinese to measure SWB and its determinants after WRH. Measurement of all variables was as follows:

#### 3.2.1. Dependent Variables: SWB

SWB has been well-researched and is a moderately stable concept [46]. There are many existing surveys focusing on SWB, including the World Values Survey (WVS), the European Quality of Life Survey (EQLS), and the European Social Survey (ESS) [30]. In this study, we referred to relevant literature [12,24,47] in our measurement of SWB on a Likert 5-point scale. Participants were asked to respond to the question: “Are you happy after WRH?”. This kind of direct collection has proven the most reliable, effective, and comparable [6,25,48].

#### 3.2.2. Independent Variables

##### Household Characteristics

Several studies have shown that the SWB of rural residents depends on their individual characteristics and their household status; it is also affected by their village environment [25,49,50,51]. Gender, education level, household living area per capita (HLAPC), age, and household income have all been shown to affect SWB [4,12,25,30,31,52].

The impact of health and social capital on the SWB of rural residents has also been evidenced [30,37]. In order to control for these impacts on SWB, we measured health and social capital on a Likert 5-point scale using the following questions: “Are you satisfied with your current health?” and “Are you satisfied with your relationships with your relatives and neighbors?”

##### Sustainability-Based Satisfaction after WRH

Drawing on the work of Kumar et al. [11], we measured residents’ satisfaction after WRH by determining their satisfaction with economic, social, environmental, and cultural factors as well as with policy. Participants were asked to respond using a Likert 5-point scale to the following questions: (1) Are you satisfied with your economic income after WRH?; (2) Are you satisfied with your access to public facilities after WRH?; (3) Are you satisfied with the overall environment of the rural village after WRH?; (4) Are you satisfied with the preservation of culture after WRH?; (5) Are you satisfied with the compensation policy for WRH?

##### WRH

The core aim of this study was to understand how differing WRH models affect the SWB of residents. Therefore, three dummy variables were used to represent the different models: dummyMode1 (index replacement, 1 = Yes, 0 = No), dummyMode2 (monetary compensation, 1 = Yes, 0 = No), and dummyMode3 (asset replacement, 1 = Yes, 0 = No). In order to further examine whether different models exert different impacts on different aspects of SWB, the dummy variables were multiplied by each of the satisfaction variables. The coefficient of the interaction term was examined to verify Hypothesis 2.

### 3.3. Estimation Strategy

Originally proposed by Theodossiou [53] and Lelkes [54], the ordered logit model [6,24] and the probit model [37,55] have become mainstream for studies exploring life satisfaction and mental health. Economists and psychologists often regard SWB as a categorical variable with a hierarchical order rather than a base variable [56]. This view favors ordinary least squares (OLS) regression estimation [30]. It has been shown that the results of the ordered model and OLS do not vary greatly [2,31,57]. This study therefore constructed an orderly probit regression of residents’ SWB after WRH:

(1) $$Y_{i}=\beta_{0}+\beta_{1}{Χ}_{1}+\cdots +\beta_{i}{Χ}_{i}+dummy_{\mathrm{mod}el1}+dummy_{\mathrm{mod}el2}+dummy_{\mathrm{mod}el3}+\varepsilon_{it}$$

where Y*_i_* represents SWB, X*_i_* represents household characteristics and satisfaction in each dimension, *β_i_* represents the coefficient of the independent variable, Dummymodel represents the dummy variables of the three models, and *ε*L represents random error and is assumed to be normally distributed, representing other unobservable factors that affect SWB, with a mean value of 0 and a variance of 1.

In order to study differences under different WRH, we generated interaction terms to distinguish the slopes of different groups. We studied the promotion effects of different WRH models using the following equation:

(2) $$Y_{i}=\rho_{0}+\rho_{0}{Χ}_{1}\times dummy_{\mathrm{mod}el1}+\rho_{1}{Χ}_{2}\times dummy_{\mathrm{mod}el2}+\rho_{1}{Χ}_{2}\times dummy_{\mathrm{mod}el2}+\rho_{2}{Χ}_{2}\times dummy_{\mathrm{mod}el2}+\cdots +\mu_{it}$$

To increase the robustness of empirical results, Knight et al. [31] suggested including explanatory variables measuring the impact of personality traits, such as income, health, and social capital. We included these in the current study but did not include motivation to participate in WRH, because the selected cases are all examples of collective voluntary participation; thus, the policy dividends provided by the government do not differ significantly.

## 4. Empirical Results

Table 2 presents descriptive statistics of the 315 samples. The majority were male (73.10%) and over 55 years old (46.03%). The education level of the respondents was mainly primary school and below (61.52%). The per capita living area of the respondents’ families was mostly (38.72%) between 44.5 m^2^ and 60 m^2^. Roughly half of the respondents (50.16%) reported themselves as “happy” following WRH, while 39.37% of the respondents rated their emotional state as “neutral”. In terms of satisfaction with the economic dimension after WRH, 46.98% of the respondents felt “satisfied”. The majority (72.7%) were also satisfied with the social dimension. In total, 53.33% and 66.03% of the respondents, respectively, expressed satisfaction with the environmental and cultural dimensions. However, 46.03% of the respondents indicated that their satisfaction level with regard to policy was “neutral”. This result is poor compared to the other dimensions.

Before formally examining the results of the ordered probit model, we must perform correlation analysis among the independent variables (see Table 3 and Table 4). We found that most of the independent variables were moderately correlated, with small correlation coefficients. That is, there was little to no overlap between the independent variables. In addition, we found that household living area per capita, income, environment, and social satisfaction were significantly correlated with SWB and that social capital was moderately correlated with SWB. Policy and income were weakly correlated with SWB. To determine whether heteroscedasticity exists in the regression, we performed the White test, with the following results: 0.0809 > 0.05. This indicates that the equation does not exhibit heteroscedasticity.

**Notes:** All of the variables were counted and calculated by the authors.

### 4.1. Determinants of SWB after WRH

This study observed the impact of satisfaction with various aspects on SWB after WRH. In all models, household variables such as education level, HLAPC, income, health, and social capital all positively impacted SWB, with the exception of gender, which exerted an insignificant negative impact (see Table 5). The pooled model (model 7) indicated that rural residents with higher HLAPC, income, or stronger social capital were happier after WRH. Observing the impact of different dimensions of satisfaction on SWB revealed that economic, social, and environmental aspects significantly enhanced SWB, with the strongest influence exerted by the environmental aspect (coefficient value of 1.229). Satisfaction with cultural preservation and compensation policy inhibited SWB; however, this was not significant. Similarly, the dummy variable representing the WRH model did not reach statistical significance. In short, the above results partially support Hypothesis 1.

### 4.2. Determinants of SWB after Different WRH

We further examined whether different WRH models promote the satisfaction of farmers and thus promote their SWB (see Table 6). As above, most of the results regarding household variables were consistent with established theory. Among them, HLAPC, income, social capital, and health exerted statistically significant impacts on SWB. The interaction terms verified Hypothesis 2; that is, satisfaction under different WRH models has differing effects on SWB. In the pooled model (Model 6), satisfaction with the economic dimension only exerted a significant effect under the index replacement model (coefficient value of 0.343). Satisfaction with economic dimension of the other two models did not reach statistical significance. Satisfaction with the social dimension significantly promoted SWB. This was confirmed in all three models. Especially in the monetary compensation mode and asset replacement mode, the positive effect of satisfaction with the social dimension on SWB was strong (coefficient values of 1.32 and 1.494, respectively). The positive effect of satisfaction with the environment on SWB was statistically significant under all models; it was strongest under the index replacement model and the monetary compensation model, and slightly weaker under asset replacement. However, satisfaction with culture and policy did not exert a statistically significant effect on SWB.

### 4.3. Robustness

In order to check the robustness of the results, we introduced instrumental variables through a simple OLS model. The instrumental variable replaces the original policy and cultural variables with “overall satisfaction with implementation of WRH (1 = very satisfied; 5 = very satisfied)”. This variable better reflects the farmers’ perception of overall village progress after WRH, meeting the requirements of “correlation” and “exogenousness” of instrumental variables. The first column of Table 7 shows that the direction of the coefficients for economic, social, and environmental factors remains significantly positive, which supports the pooled result in Table 5. We also drew on the work of Liang et al. [58] and reduced the total number of samples from 315 to 285 through bilateral shrinking of the 5% quantile. The second column of Table 7 shows that coefficients of all variables remained unchanged and reached significance.

## 5. Discussion

Although the SWB of land-lost farmers has been well-researched over the years [9,11,12,21], there exists a gap with regard to the impact of satisfaction with various aspects on SWB following the implementation of WRH policy in China. A comparison of the differences between different exit modes has also been lacking. The current paper fills these gaps and makes novel contributions to this research topic.

We found that the basic household characteristics of income, health, household living area per capita, and social capital have a significant positive impact on SWB in most cases. This echoes the literature, which offers evidence regarding the effects of income [31,48], health [12], and social capital [37]. The significant effect of household living area per capita on SWB not only solves the problems of dilapidated houses and earthquake resistance in the currency compensation model, but it also solves the “one household with multiple houses” in the asset replacement model and the “one house with multiple households” in the index replacement model. The improvement in PCLS is significant to the development of individual farmers as the new generation moves out from older houses to new houses to increase their quality of life. This supports previous findings that report improvements to housing conditions being positively linked to SWB [59,60].

The pooled results indicated that satisfaction with economic, social, and environmental factors after WRH positively impacts SWB, which validates Hypothesis 1. However, in-depth analysis of different WRH models reveals that the impact of various aspects on SWB under different models varies, which supports Hypothesis 2. The significant positive impact of economic satisfaction on SWB was only evident in the index replacement model, which may be attributed to the village collective’s financial guarantee of the livelihoods of residents after reconstruction and reasonable compensation mechanisms [16]. In this model, residents voluntarily participate in transformation, and the village collective does not requisition farmers’ farmland; therefore, agricultural production is protected. The collective economic model formed in the countryside also enables farmers with relatively low income to obtain “policy dividends” faster and improve their quality of life within a short period of time, while farmers with relatively strong economic strength become the engine that drives the village’s collective economy. Most of the resettlement houses in this model are planned on state-owned land, and the certification of property rights for resettlement housing has been perfected. Therefore, resettlement houses with ownership certificates can be circulated in the market, which greatly improves the economic value of housing. This finding supports the results of Patil et al. [61], who researched preferences among lost-land farmers in India and found that compensation options such as land holding, employment, and guarantees of sustainable livelihoods are better choices, compared with pure monetary compensation.

Satisfaction with social and environmental factors has a significant positive impact on SWB in all models, which is in line with the argument that the improvement of public facilities and services is a necessary condition for improving SWB [6,11,62]. For example, in the index replacement model, Dapu Village re-planned the original village through the village collective and built various public facilities such as elderly activity centers, primary schools, and health centers. Redundant land was also used for the construction of parks and gymnasiums. The Guishan community, under the asset replacement model, moved to a well-planned community after WRH and transformed from a rural household registration to an urban household registration. This example echoes the finding of Liang and Zhu [9], who found the existence of a positive connection between social security and SWB. From the perspective of resettlement compensation, the demolition and reconstruction of Guishan Village (i.e., the asset replacement model) enabled the collective to take care of vulnerable groups.

Viganó et al. [27] pointed out that cultural factors had a weak impact on the SWB of rural residents in Italy. Similarly, in this study, we found that satisfaction with cultural preservation had no significant impact on SWB under all models. This may be due to rapid urbanization, coupled with the consideration of the overall environmental improvement. Most rural areas are introducing green approaches to traditional funeral and wedding rituals, which diminishes the importance of culture in these communities. Zhang and Qian [20] found that older villagers are more likely to hold fast to cultural traditions, especially rural residents. We further found that satisfaction with policy has no significant impact on SWB. This seems to reflect the dissatisfaction of residents with housing compensation, implying that compensation policy is a key factor in decision-making regarding WRH [13,16,63]. Under the asset replacement model, which implemented different compensation policies in different periods of promotion, some residents became psychologically imbalanced. In the monetary compensation model, once residents had chosen monetary compensation, they no longer enjoyed the right to distribute the benefits of the homestead, which in turn inhibited their happiness. No matter which model is selected, decision-makers should carefully consider how to integrate traditional culture with development to increase the participation of local residents, as suggested by Viganó et al. [27]. Moreover, supporting cultural adaptability is essential, particularly for transformations to urban household registration [20]. It seems necessary for SWB that residents develop a deep understanding of the measures, benefits, and promotion procedures of WRH and that policy remains consistent in the various stages of promotion.

## 6. Conclusions

As an important policy tool focused on the improvement of vacant rural land in China, WRH policy has a multi-dimensional impact on rural villages in terms of economic, social, and environmental factors. These aspects further impact the SWB of residents. This study surveyed 315 residents from four rural areas of Jinjiang City subjected to three WRH models. The pooled empirical results point out that satisfaction with economic, environmental, and social factors makes a significant positive impact on SWB. Interaction terms indicated that residents in the index replacement model experienced increased SWB from economic, environmental, and social factors, while residents in the asset replacement and monetary compensation model experienced positive impacts only in terms of environmental and social factors. Household characteristics such as income, health, household living area per capita, and social capital all positively impacted SWB, which supports the findings in the literature. However, the fact that satisfaction with cultural and policy factors had no impact reveals the shortcomings of current WRH policy.

The results from this study provide evidence that the WRH policy can promote the SWB of residents from the perspective of sustainable development. They also have reference and policy implications for China’s comprehensive expansion of WRH policy and for other developing countries implementing similar policies. Although all three models reflect positive effects in terms of social and environmental factors on SWB, the index replacement model proved the most effective. This model not only implements the protection of property rights for resettlement houses, but it also avoids the expropriation of agricultural land, creating more security in terms of sustainable livelihoods. Although under the asset replacement model farmers receive houses in the same area, these cannot be traded within the existing legal system. Therefore, villagers who receive compensation should receive certification of house property rights. In addition, under the monetary compensation model, farmers can only receive a one-time monetary compensation, and it is necessary to make clear and reasonable regulations regarding the flow of funds to avoid the loss of farmers’ rights when compensation is not received. This study also suggests that whatever WRH model is implemented, farmers’ understanding and participation in the WRH policy should be strengthened. Moreover, local cultural elements and characteristics should be better integrated into planning and design.

Despite its valuable contributions, this study is subject to several limitations. First, comparative studies on the differences in satisfaction over time and their impact on happiness are required. Second, due to limited resources, the sample size was restricted to 300 respondents. This could be expanded in future research to increase reliability and generalizability. Subsequent research could also use the natural experiment method and other means to further explore differences in SWB and their determinants in other rural locations under WRH policy.

## Figures and Tables

**Table 1:** Descriptions of study areas.

WRH Model	Area	Village Characteristics	Degree of Policy Implementation	Difficulties in Policy Implementation
Index replacement model	Qiekeng	The total land area of the village is 1.8893 km². The area of rural settlements is 0.5553 km², and the cultivated land area is 0.9067 km². There are 3979 registered residence households and 951 households in the village. The villagers are mainly engaged in international business, and the collective economy of the village is relatively weak.	Village reconstruction was completed in 2019.	Due to economic conditions, the villagers could not participate in the transformation.
	Dapu	The total land area is 3 km², with 5046 registered residents and 3500 immigrants. Before the reconstruction, the village environment was dirty and neglected, with low living standards.	By 2020, 80% of the new housing construction had been completed.	Due to the insufficient balance of distributable indicators, the villagers who participated in the exit wanted to plan the village, resulting in stagnation of the task.
Asset replacement model	Guishan	The population of the community is 3,412,881, with more than 3500 overseas Chinese relatives living abroad, covering an area of 2 km². Before the transformation, there were more than 1500 houses in the original Guishan Village, with multiple families per household. There was a large number of uninhabited ancestral houses in the original village collective.	In 2014, the government completed the transformation of the village and the relocation of the residents to the city.	Because of the location of resettlement houses in the city, farmers lost their livelihood after the transformation.
Monetary compensation model	Xibian	The village has a population of more than 1000 people, residing in about 260 households. There are about 140 houses in the village. These houses were built many years ago and are mostly dilapidated.	Of the more than 100 households in the old village reconstruction area, about 40 to 50 households already have houses.	At present, the biggest difficulty in promoting the project is that the new housing base is insufficient and farmers are not willing to live in high-rise apartments.

**Notes:** All of the information is collected by the authors.

**Table 2:** Descriptive statistics of valid samples.

Measurement	Item	Proportion	Sample	Definition
Happiness				
SWB	Not happy at all	1.27	4	SWB of respondents
	Not happy	4.76	15	
	So-so	39.37	124	
	Happy	50.16	158	
	Very happy	4.44	14	
Variables for household characteristics				
Age	1–25	1.9	6	Age of respondents
	26–40	15.24	48	
	41–55	36.83	116	
	>55	46.03	145	
Gender	Male	73.33	231	Personality of respondents
	Female	26.67	84	
Education	Illiterate	30.79	97	Education level of respondents
	Primary school	30.79	97	
	Junior high school	13.66	43	
	Above senior high school	24.76	78	
HLAPC	<33.5 m^2^	22.23	70	Household living area per capita
	33.5–44.5 m^2^	19.68	62	
	44.5–60 m^2^	38.72	122	
	>60 m^2^	19.37	61	
Revenue	Less than 80,000 yuan	18	5.71	Respondents’ annual household income
	RMB 80,000–100,000	40	12.7	
	RMB 100,000–150,000	126	40	
	RMB 150,000–200,000	109	34	
	More than 200,000 yuan	22	6.98	
Health	Very unhealthy	0.32	1	Respondents’ self-rated health status
	Unhealthy	2.22	7	
	Just fine	22.22	70	
	Healthy	69.52	219	
	Very Healthy	5.72	18	
Social Capital	Very dissatisfied	0	0	Respondents rated their own social capital
	Dissatisfied	4.76	15	
	Neutral	37.47	118	
	Satisfied	55.87	176	
	Very satisfied	1.9	6	
Variable for sustainability-based satisfaction after WRH				
Satisfaction with economic	Very dissatisfied	0.63	2	Respondents’ satisfaction with their economic income
	Dissatisfied	6.35	20	
	Neutral	40.32	127	
	Satisfied	46.98	148	
	Very satisfied	5.72	18	
Satisfaction with social	Very dissatisfied	0	0	Respondents’ comments on construction of public facilities
	Dissatisfied	0	0	
	Neutral	16.19	35	
	Satisfied	72.7	229	
	Very satisfied	11.11	51	
Satisfaction with culture	Very dissatisfied	0	0	Respondents’ comments on the cultural atmosphere of the village
	Dissatisfied	1.59	5	
	Neutral	32.7	103	
	Satisfied	53.33	168	
	Very satisfied	12.38	39	
Satisfaction with environment	Very dissatisfied	0	0	Respondents’ overall evaluation of the village
	Dissatisfied	1.59	5	
	Neutral	28.25	89	
	Satisfied	66.03	208	
	Very satisfied	4.13	13	
Satisfaction with WRH policy	Very dissatisfied	2.54	8	Respondents’ comments on compensation policies
	Dissatisfied	10.16	32	
	Neutral	46.03	145	
	Satisfied	35.87	113	
	Very satisfied	5.4	17	
Variables for WRH				
Mode	Index replacement model	60.64	191	Homestead postponement mode selected by respondents
	Asset replacement model	19.68	62	
	Monetary compensation model	19.68	62	

**Table 3:** Pearson correlation matrix for theoretical variables.

	Age	Gender	Education	HLAPC	Economic	Culture	Social	Environment	Policy	Social Capital	Revenue	Health
Age	-											
Gender	0.1799	-										
Education	−0.4871	−0.1149	-									
HLAPC	−0.0345	0.0617	0.0992	-								
Economic	−0.0732	0.0164	0.1517	0.1743	-							
Culture	−0.0121	−0.0184	0.0051	−0.1189	−0.0284	-						
Social	0.1331	0.1295	0.0204	0.2121	0.2484	−0.0483	-					
Environment	−0.013	0.0522	0.1244	0.147	0.0287	−0.0436	0.416	-				
Policy	−0.0182	0.0035	0.0665	−0.0836	0.1415	−0.1123	−0.0273	0.0071	-			
Social Capital	−0.0802	−0.0239	−0.1159	0.0035	−0.0011	0.0609	−0.1364	−0.2252	−0.0965	-		
Revenue	−0.0412	0.0189	0.1182	0.0447	0.5041	−0.0923	0.2878	0.0474	0.1838	0.026	-	
Health	0.0316	−0.0292	0.0311	−0.0445	0.1038	−0.0017	0.1297	0.0304	−0.0673	−0.0568	0.1337	-

**Notes:** All of the variables were counted and calculated by the authors.

**Table 4:** Correlation between dependent and independent variables.

Variables	Pearson Correlation	Variables	Pearson Correlation	Variables	Pearson Correlation
Gender	0.0543	Environment	0.437 ***	Social	0.372 ***
Age	−0.0612	Policy	−0.0627 *	Economic	0.0801 *
Education	0.0103	HLAPC	0.00937 ***	Health	0.00403
Culture	−0.0199	Revenue	0.163 ***	Social Capital	0.132 **

Notes: *, **, and *** represent significance at the 10%, 5%, and 1% levels, respectively. Robust standard errors are shown in parentheses. All of the variables were counted and calculated by the authors.

**Table 5:** Pooled results (dependent variables: SWB).

Variable	(1)	(2)	(3)	(4)	(5)	(6)	(7)
Age	−0.0513	−0.0460	−0.188 *	−0.0594	−0.0507	−0.0553	−0.160
	(0.0982)	(0.0984)	(0.104)	(0.104)	(0.0982)	(0.0983)	(0.109)
Gender	0.247	0.247	0.136	0.212	0.247	0.250 *	0.141
		(0.151)	(0.159)	(0.159)	(0.151)	(0.151)	(0.166)
	(0.151)						
Education	0.0634	0.0580	0.0629	0.0224	0.0641	0.0655	0.0252
	(0.0498)	(0.0501)	(0.0521)	(0.0526)	(0.0499)	(0.0499)	(0.0550)
HLAPC	0.0303 ***	0.0293 ***	0.0266 ***	0.0305 ***	0.0301 ***	0.0297 ***	0.0267 ***
	(0.00433)	(0.00437)	(0.00464)	(0.00463)	(0.00435)	(0.00436)	(0.00498)
Log Revenue	0.491 ***	0.432 ***	0.359 ***	0.615 ***	0.489 ***	0.498 ***	0.448 ***
	(0.0760)	(0.0833)	(0.0811)	(0.0830)	(0.0761)	(0.0764)	(0.0949)
Health	0.126	0.116	0.0233	0.136	0.128	0.109	0.0252
	(0.112)	(0.112)	(0.118)	(0.119)	(0.112)	(0.113)	(0.126)
Social Capital	−0.0749	−0.0839	0.132	0.163	−0.0733	−0.0707	0.289 *
	(0.134)	(0.135)	(0.144)	(0.145)	(0.135)	(0.135)	(0.153)
Mode2	0.289	0.327 *	0.0504	0.531 ***	0.299	0.208	0.291
	(0.181)	(0.183)	(0.193)	(0.195)	(0.182)	(0.196)	(0.226)
Mode3	0.112	0.0975	0.0333	−0.0267	0.110	0.128	−0.0787
	(0.178)	(0.179)	(0.189)	(0.191)	(0.179)	(0.179)	(0.202)
Income		0.188 *					0.226 *
		(0.107)					(0.120)
Social			1.231 ***				0.960 ***
			(0.142)				(0.158)
Environment				1.410 ***			1.229 ***
				(0.156)			(0.173)
Culture					−0.0465		−0.0549
					(0.0987)		(0.109)
Policy						−0.0986	−0.104
						(0.0905)	(0.101)
Obs.	315	315	315	315	315	315	315

Notes: *, and *** represent significance at the 10% and 1% levels, respectively. Robust standard errors are shown in parentheses. All of the variables were counted and calculated by the authors.

**Table 6:** Index system for variable cross-items.

Variable	(1)	(2)	(3)	(4)	(5)	(6)
Age	−0.0357	−0.190 *	−0.0640	−0.0332	−0.0491	−0.136
	(0.0972)	(0.103)	(0.103)	(0.0973)	(0.0977)	(0.112)
Gender	0.251 *	0.133	0.204	0.242	0.250 *	0.0884
	(0.150)	(0.158)	(0.159)	(0.150)	(0.15)	(0.169)
Education	0.0666	0.0584	0.0191	0.0741	0.0718	0.0320
	(0.0493)	(0.0513)	(0.0519)	(0.0491)	(0.0491)	(0.0555)
HLAPC	0.0289 ***	0.0270 ***	0.0301 ***	0.0300 ***	0.0293 ***	0.0263 ***
	(0.00434)	(0.00462)	(0.00460)	(0.00434)	(0.00438)	(0.00519)
Log Revenue	0.437 ***	0.374 ***	0.631 ***	0.493 ***	0.494 ***	0.434 ***
	(0.0834)	(0.0804)	(0.0821)	(0.0750)	(0.0755)	(0.0996)
Health	0.121	0.0238	0.130	0.131	0.107	6.961 ***
	(0.112)	(0.118)	(0.119)	(0.112)	(0.114)	(1.25)
Social Capital	−0.0823	0.119	0.160	−0.0887	−0.0602	8.614 ***
	(0.134)	(0.143)	(0.144)	(0.136)	(0.135)	(1.286)
Economic*Mode1	0.162 *					0.343 **
	(0.107)					(0.15)
Economic*Mode2	0.253 **					0.0490
	(0.118)					(0.233)
Economic*Mode3	0.197 *					0.0312
	(0.111)					(0.303)
Social*Mode1		1.195 ***				0.817 ***
		(0.141)				(0.179)
Social*Mode2		1.247 ***				1.312 ***
		(0.143)				(0.350)
Social*Mode3		1.218 ***				1.494 ***
		(0.143)				(0.389)
Environment*Mode1			1.355 ***			1.288 ***
			(0.153)			(0.205)
Environment*Mode2			1.525 ***			1.338 ***
			(0.168)			(0.342)
Environment*Mode3			1.351 ***			0.949 ***
			(0.153)			(0.334)
Cultural*Mode1				−0.0751		−0.0990
				(0.100)		(0.129)
Cultural*Mode2				0.00914		−0.0657
				(0.100)		(0.243)
Cultural*Mode3				−0.0236		0.106
				(0.107)		(0.266)
Policy*Mode1					−0.142 *	0.0191
					(0.0841)	(0.131)
Policy*Mode2					−0.111	−0.222
					(0.109)	(0.165)
Policy*Mode3					−0.0984	−0.216
					(0.0896)	(0.334)
Observation	315	315	315	315	315	315

Notes: *, **, and *** represent significance at the 10%, 5%, and 1% levels, respectively. Robust standard errors are shown in parentheses. All of the variables were counted and calculated by the authors.

**Table 7:** Result of robustness check.

Variables	(1)	(2)
Environment	0.975 ***	0.904 ***
	(0.186)	(0.185)
Social	0.808 ***	0.985 ***
	(0.177)	(0.168)
Economic	0.510 ***	0.266 **
	(0.123)	(0.132)
Satisfaction (Instrumental variable)	0.494 ***	
	(0.0522)	
Cultural		−0.0166
		(0.124)
Policy		−0.109
		(0.120)
Control variables	Yes	Yes
Observations	315	285
Type of method	OLS	Probit

Notes: **, and *** represent significance at the 5%, and 1% levels, respectively. Robust standard errors are shown in parentheses. All of the variables were counted and calculated by the authors.

## Data Availability

Not applicable.
